# Galilei Corneal Tomography for Screening of Refractive Surgery Candidates: A Review of the Literature, Part II

**Published:** 2019

**Authors:** Majid Moshirfar, Mahsaw N. Motlagh, Michael S. Murri, Hamed Momeni-Moghaddam, Yasmyne C. Ronquillo, Phillip C. Hoopes

**Affiliations:** 1 Department of Ophthalmology and Visual Sciences, John A. Moran Eye Center, School of Medicine, University of Utah Salt Lake City, UT, USA; 2 HDR Research Center, Hoopes Vision, Draper, UT, USA; 3 Department of Ophthalmology, College of Medicine, University of Arizona, Tucson, AZ, USA; 4 Department of Optometry, School of Paramedical Sciences, Mashhad University of Medical Sciences, Mashhad, Iran

**Keywords:** Corneal Biomechanics, Ectasia, Refractive Surgery, Pentacam; Galilei, Corvis, Keratoconus, Scheimpflug Imaging

## Abstract

Corneal topography is the most widely used technology for examining the anterior corneal surface. Scheimpflug imaging is a newer technique that allows for measurement of both the anterior and posterior corneal surface, which allows for three-dimensional reconstruction of the cornea. This is of particular interest and value in the field of cataract and refractive surgery. The Galilei camera is a commercially available dual Scheimpflug system that combines curvature data from Placido disc-based corneal topography with elevation data from Scheimpflug technology. The addition of Placido disc topography makes the Galilei unique from its more popular counterpart, the Pentacam, which was discussed in Part I. Compared to the Pentacam, and however, the Galilei analyzer is a newer system that has emerged as a valuable screening tool given its dual Scheimpflug capability. In the first article of this series, the authors summarized the refractive indices available on the Pentacam system with the purpose of identifying the best diagnostic parameters for keratoconus. Similarly, the purpose of this article is to summarize corneal surface indices available on the Galilei system and evaluate their use in screening of the refractive surgery candidate. Since post-operative keratectasia is still prevalent, this paper aims to identify the most clinically relevant indices that may be used in pre-operative evaluation.

## INTRODUCTION

**Function of Dual Scheimpflug Imaging**

As discussed in Part I of this article series, anterior segment imaging has drastically changed over the last 25 years. Since its introduction in the 1980s, topography has vastly altered the sphere of pre-operative screening for refractive surgery candidates [1, 2]. Tomographic devices have many functions including evaluation of cataracts, calculations of intraocular lenses (IOLs) power, guiding of surgical plans, and longitudinal follow-up of patients with known disease. In the realm of refractive surgery, the fundamental purpose of any tomographic device is to appropriately screen surgical candidates and facilitate high quality outcomes for patients. In this review, we focus on the Galilei dual Scheimpflug system (Ziemer Ophthalmic Systems AG, Port, Switzerland), which is one of the newer tomography devices available for refractive and cataract surgery. The Galilei analyzer is a uniquely elegant system that combines corneal curvature data from Placido disc topography with elevation data from Scheimpflug technology. The device itself has a rotating camera head that can capture two Scheimpflug slit images and Placido disc simultaneously [3, 4]. By combining height data from the slit images, the camera can track decentration from the inevitable micromovements of the eye. A benefit of the Galilei compared to other Scheimpflug analyzers such as Pentacam (Oculus Optikgeraete GmbH; Wetzlar, Germany) or Orbscan II (Bausch & Lomb, Orbtek Inc., Utah, USA) is its ability to reduce motion error by correcting eye motion and cyclotorsion through a software algorithm. Moreover, height data from both Scheimpflug and Placido are fitted to the anterior corneal surface data [5]. Data for the posterior corneal surface is derived from the images of the dual Scheimpflug analyzer. Another advantage of the Galilei comes in the form of corneal aberration analysis. The system calculates wavefront aberrations for both the anterior and posterior corneal surface independent of aberrations from the lens [1]. This data is then displayed alongside root mean square indices calculated over a 6.0-mm optical zone centered over the pupil [1]. 

When compared to other tomography devices, dual Scheimpflug imaging improves posterior corneal measurements and provides for excellent accuracy in assessing corneal thickness [6-12]. Moreover, from a practical standpoint, the Galilei can perform all the measurements a refractive surgeon may require prior to surgery. The convenience of having this “one-stop shop” is advantageous to the patient experience and for clinical workflow. Another benefit of the Galilei is calculation of total corneal power using ray tracing, which refracts incoming parallel rays through the anterior and posterior corneal surface. The calculated power and axis of astigmatism from these measurements are immensely helpful in pre-operative planning of IOLs and limbal relaxing incisions. Studies have shown that Galilei has superior IOL power prediction with a lower error when compared to other Scheimpflug devices [13, 14]. Despite demonstrating superior repeatability in thickness and higher order aberrations [14-20], there is less data available for the Galilei because it is a newer system. This is a limitation as longitudinal analysis, and further repeatability studies are required to ascertain the actual utility of the Galilei device in clinical practice. 

**Application for Screening Refractive Surgery Candidates**

The Galilei device offers advantages in both accuracy and reproducibility that are valuable in understanding the corneal surface [7, 15, 21, 22]. Galilei has the highest level of repeatability compared to other Scheimpflug systems [23]. As a dual Scheimpflug analyzer, the Galilei eliminates the parallax of a single image and compensates for off-center measurements. Thus, theoretically, the Galilei camera can provide higher sensitivity and specificity in detecting early ectatic changes on the corneal surface. When considering refractive screening indices, anterior corneal measurements from Placido imaging aid in improving sensitivity, while the posterior measurements derived from Scheimpflug imaging improve specificity. By integrating these data points, the Galilei has the unique potential of becoming an excellent screening tool in clinical practice. 

As discussed in Part I of this article series, the age-old question remains: what are we screening for? While many classification systems for keratoconus (KC) exist [24-33], the community of refractive surgeons has failed to agree on a universal definition of early ectasia [4]. This history of this debate is discussed at length in Part I of this article series. As such, we continue to use pre-keratoconus as a unified term that includes form-fruste KC, subclinical KC, borderline KC, suspect KC, and early KC. In this article, we present a review of the literature as it pertains to the Galilei system and highlight the clinical application of screening indices in discerning normal and ectatic corneas.

## METHODS

A literature review was performed using various databases, including PubMed, Mendeley, Ovid, Elsevier, and Science Direct. For the database search the primary search term included “Galilei”, which was connected to descriptors such as “LASIK”, “progression parameters”, “screening”, “comparison”, “dual Scheimpflug”, “tomography”, “topography”, “keratoconus”, “subclinical keratoconus”, “evaluation”, “G6”, “G4”, “G2”, “analyzer”, “index”, “indices” and various others. Peer-reviewed and scholarly resources, including original scientific articles as well as review articles were included. Publications between 1900 and 2019 were included in this review. Articles were screened for relevance and significance based on their abstracts. Those that were identified as appropriate for this review were included. Additional searches were made to find relevant literature through Mendeley, Ovid, Elsevier, and ScienceDirect. All articles that were deemed relevant to this topic were included in this review.

As described in Part I, studies that evaluate refractive indices rely on the area under the curve (AUC) to determine diagnostic accuracy. Therefore, Galilei parameters with AUC >0.900 were judged suitable for screening of KC, while parameters with AUC >0.800 were selected for screening of pre-keratoconus. The selected parameters that met these requirements were then incorporated in creating proposed cut-off thresholds. Indices that met these criteria in at least two studies were then averaged based on the cut-off value proposed by the individual study. Clinically accepted normal index ranges for the parameters evaluated in this article are shown in Table 1. In Table 2 the highlighted parameters indicate the selected cut-off values that were averaged for screening of KC. However, given the limited studies evaluating Galilei with cases of pre-keratoconus, we elected to highlight the parameters with an AUC >0.800 in Table 3 as there was insufficient data to develop proposed thresholds.

**Indices for Refractive Screening**

Like other corneal imaging systems, the Galilei device has indices for topographic, topometric, pachymetric, and corneal wavefront data. Our focus is to define and review the specific keratoconus indices included in Galilei software that are most valuable to clinical practice. A unique feature of the Galilei system is that it automatically evaluates certain parameters in the prediction of KC. Galilei’s output reports are extremely user friendly and include the Refractive Report, the Keratoconus Report, the Wavefront Report, and the IOL Power Report [2]. While no formal thresholds have been set to define abnormal, we have provided the clinically accepted normal index ranges in Table 1. Based on the available literature, a side-by-side comparison of screening indices along with their respective sensitivity, specificity, and area under the curve (AUC) for both clinical and subclinical KC can be found in Table 2 and Table 3 respectively. A simplified version of these screening indices along with our recommended cut-off values is found in Table 4. 

The following subsections detail the several indices available through the Galilei system for screening. As mentioned previously, there are few investigations of Galilei as it is a newer imaging system. Cognizant of this, we encourage the clinician to pay special attention to the following studies as they were best in evaluating the Galilei device based on selection criteria, methods, and study size: Shetty *et al* (number [n] = 42 normal, 51 KC, 37 pre-keratoconus) [3], Feizi et al (n = 136 normal, 51 KC, 23 pre-keratoconus) [4], Smadja et al (n = 177 normal, 148 KC, 47 pre-keratoconus) [5], Demir et al (n = 151 normal, 67 KC) [6], and the recent study by Golan et al (n = 178 normal, 31 pre-keratoconus) [7].

Throughout each subsection, the clinician is encouraged to keep in mind that although almost all indices are validated for detection of KC, the opposite holds true in the discrimination of pre-keratoconus. Moreover, based on the aforementioned studies, we recommend the following indices as the most effective for detection of pre-keratoconus and recommend the clinician’s special attention to asphericity asymmetry index (AAI) and surface regularity (SRI). While AAI lacks published values demonstrating its diagnostic accuracy as assessed by AUC, it demonstrates superior sensitivity and specificity that warrants its consideration for pre-keratoconus scrrening. Moreover, based on our review of the literature, standard deviation of corneal power (SDP) demonstrated the single highest AUC for detection of pre-keratoconus (Table 3). 

**Table 1:** Accepted Abnormality Thresholds of Keratoconus Indices for the Galilei System Based on Literature Review

Parameter	Description	Abnormal Threshold
AAI	Asymmetry of asphericity over the corneal surface correlates to rate of curvature change	> 25
CSI	Difference between the area-corrected corneal power between two rings on the corneal surface	> 1.00
DSI	Greatest difference between any two 45-degree corneal sectors	> 3.50
IAI	Average of corneal power variations along each meridian of the corneal surface	> 0.50
I-S	Dioptric asymmetry between the inferior and superior corneal hemispheres	> 1.4
KPI	Compilation index that describes the percent probability of keratoconus	> 30
OSI	Greatest difference between any two oppositely positioned 45-degree corneal sectors	> 2.10
PPK	Probability index that describes the optimal threshold for detecting keratoconus	> 45
SDP	Standard deviation of total corneal power	> 2.00
SAI	Average of differences in corneal power between opposite points on the corneal surface	> 0.50
SRI	Sum of power variations along ten central rings over corneal surface, characterizes local fluctuations	> 1.55

Abbreviations: AAI: Asphericity Asymmetry Index; CSI: Center/Surround Index; DSI: Differential Sector Index; I-S: Inferior-Superior Index; IAI: Irregular Astigmatism Index; KPI: Keratoconus Probability Index; SDP: Standard Deviation of Corneal Power; OSI: Opposite Sector Index; PPK: Percentage Probability of Keratoconus; SAI: Surface Asymmetry Index; SRI: Surface Regularity Index.

**Keratoconus Predictive Indices**

***Asphericity Asymmetry Index***

**Figure 1:**
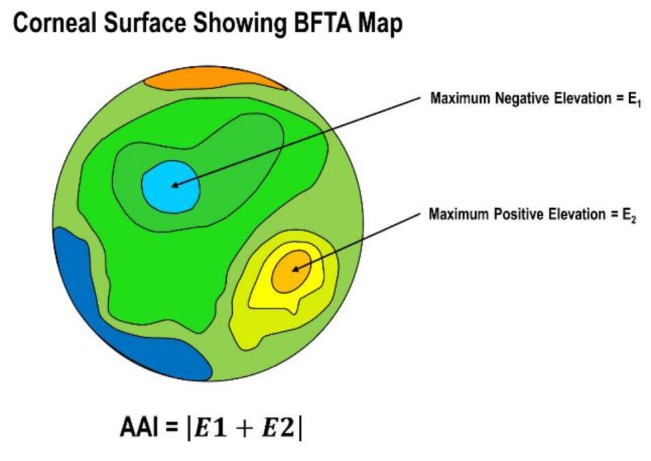
The Asphericity Asymmetry Index (AAI) Quantifies the Asymmetry of Asphericity over the Corneal Surface. As Shown by Smadja et al. [5, 11], it is Best Assessed using the Best-fit Toric Aspheric (BFTA) Maps of the Galilei System. AAI is Calculated as the difference between the Maximum Negative and Positive BFTA elevation Values

The asphericity asymmetry index (AAI), also known as the Kranemann-Arce index, is a parameter that quantifies the asymmetry of asphericity over the corneal surface [8]. The AAI is calculated as the magnitude of difference between the maximum negative best-fit toric aspheric (BFTA) reference surface value and the maximum positive BFTA elevation value (Fig. 1) [5, 8]. Symmetric aspheric meridians have elevation values closer to zero, while asymmetric aspheric meridians have higher variability in the change of curvature within each hemimeridian. 

**Table 2:** Summary of Galilei Keratoconus Parameters in Detecting Clinical Keratoconus

Study	Cut-off Value	Sensitivity	Specificity	AUC
AAI				
**Smadja et al [** 5 **]**	34.5	1.000	0.9995	-
CLMI				
**Shetty et al [** 34 **]**	-	-	-	0.966
**Mahmoud et al [** 48 **]**	-	0.892	0.988	0.950
**Mahmoud et al [** 37 **]**	-	0.994	0.996	0.995
**Kocamiş et al [** 39 **]**	1.82	0.890	0.940	0.920
CSI				
**Demir et al [** 35 **]**	-	-	-	0.918
**Feizi et al [** 36 **]**	0.700*	0.920	0.950	0.951
**Shetty et al [** 34 **]**	0.700	0.135	0.143	0.906
DSI				
**Demir et al [** 35 **]**	-	-	-	0.989
**Feizi et al [** 36 **]**	3.26	0.980	0.910	0.977
**Shetty et al [** 34 **]**	3.26*	0.904	0.976	0.983
IAI				
**Demir et al [** 35 **]**	-	-	-	0.960
**Feizi et al [** 4 **]**	0.580*	0.920	0.920	0.974
**Shetty et al [** 3 **]**	0.580	0.673	1.000	0.973
I-S				
**Demir et al [** 6 **]**	-	-	-	0.968
**Feizi et al [** 4 **]**	2.33	0.900	0.980	0.971
**Shetty et al [** 3 **]**	2.33*	0.885	0.952	0.980
KPI				
**Feizi et al [** 4 **]**	18.55*	1.000	0.990	0.999
**Shetty et al [** 3 **]**	18.55	0.962	0.952	0.993
**Mahmoud et al [** 37 **]**	-	0.850	1.000	-
Kprob				
**Feizi et al [** 4 **]**	25.55*	0.980	0.990	0.998
**Shetty et al [** 3 **]**	25.55	0.962	0.952	0.993
OSI				
**Demir et al [** 6 **]**	-	-	-	0.987
**Feizi et al [** 4 **]**	2.04*	0.960	0.990	0.988
**Shetty et al [** 3 **]**	2.04	0.923	0.952	0.983
PPK				
**Shetty et al** [3]	45.0	1.000	0.286	0.968
SAI				
**Demir et al [** 6 **]**	-	-	-	0.998
**Feizi et al [** 4 **]**	1.25*	1.000	0.990	0.999
**Shetty et al [** 3 **]**	1.25	0.923	0.952	0.984
SDP				
**Feizi et al [** 4 **]**	1.93*	0.920	1.000	0.993
**Shetty et al [** 3 **]**	1.93	0.981	0.833	0.986
SRI				
**Demir et al [** 6 **]**	-	-	-	0.942
**Feizi et al [** 4 **]**	1.52	0.900	1.000	0.982
**Shetty et al [** 3 **]**	1.52*	0.654	1.000	0.992
TCP-Central				
**Reddy et al [** 32 **]**	45.40	0.870	0.960	0.940
**Demir et al [** 6 **]**	-	-	-	0.984
**Feizi et al [** 38 **]**	45.69*	0.894	0.995	0.986
TCP-Flat				
**Reddy et al [** 32 **]**	44.50	0.690	0.990	0.790
**Feizi et al [** 38 **]**	44.04*	0.732	0.980	0.915
TCP-Steep				
**Reddy et al [** 32 **]**	46.10	0.980	0.970	0.990
**Feizi et al [** 38 **]**	46.47*	0.944	0.990	0.994
Total HOA				
**Kocamiş et al [** 39 **]**	0.873	0.840	0.810	0.820
Total RMS				
**Reddy et al [** 32 **]**	2.05	0.920	0.910	0.970
Trefoil Z (3, -3)				
**Reddy et al [** 32 **]**	0.31	0.500	0.980	0.700
Vertical Coma				
**Reddy et al [** 32 **]**	-1.07	0.630	1.000	0.800
**Kocamiş et al [** 39 **]**	-0.312*	0.850	0.840	0.850
3rd-order RMS				
**Reddy et al [** 32 **]**	1.1	0.810	0.980	0.910
4th-order RMS				
**Reddy et al [** 32 **]**	0.47	0.820	0.950	0.930
5th-order RMS				
**Reddy et al [** 32 **]**	0.15	0.840	0.940	0.900
6th-order RMS				
**Reddy et al [** 32 **]**	0.06	0.820	0.900	0.890

Abbreviations: AAI: Asphericity Asymmetry Index; AUC: Area under the Curve; CLMI: Cone Location and Magnitude Index; CSI: Center/Surround Index; DSI: Differential Sector Index; HOA: Higher Order Aberration; I-S: Inferior-Superior Index; IAI: Irregular Astigmatism Index; KPI: Keratoconus Probability Index; Kprob: Keratoconus Probability; OSI: Opposite Sector Index; PPK: Percentage Probability of Keratoconus; TCP: Total Corneal Power; RMS: Root Mean Square SAI: Surface Asymmetry Index; SDP: Standard Deviation of Corneal Power; SRI: Surface Regularity Index. *denotes cut-off point with best area under the curve if more than one study evaluated index accuracy. Highlighted indices represent the parameters that were included in the final evaluation of proposed thresholds.

**Table 3:** Summary of Galilei Keratoconus Parameters in Detecting Pre-Keratoconus

Study	Cut-off Value	Sensitivity	Specificity	AUC
AAI				
**Smadja et al [** 5 **]**	21.5	0.936	0.972	-
**Golan et al [** 7 **]**	13.5	0.774	0.670	0.750
CLMI				
**Shetty et al [** 3 **]**	-	-	-	0.314
CSI				
**Feizi et al [** 4 **]**	0.99	0.217	1.000	0.534
**Shetty et al [** 3 **]**	0.90*	0.973	0.047	0.556
DSI				
**Feizi et al [** 4 **]**	1.725*	0.739	0.556	0.646
**Shetty et al [** 3 **]**	1.730	0.541	0.698	0.627
IAI				
**Golan et al [** 7 **]**	0.400	0.516	0.736	0.625
**Feizi et al [** 4 **]**	0.445	0.739	0.541	0.664
**Shetty et al [** 3 **]**	0.450*	0.541	0.814	0.858
I-S				
**Feizi et al [** 4 **]**	1.60*	0.348	0.895	0.597
**Shetty et al [** 3 **]**	1.60	0.108	0.907	0.595
KPI				
**Feizi et al [** 4 **]**	5.00*	0.565	0.835	0.710
**Shetty et al [** 3 **]**	5.00	0.568	0.581	0.629
Kprob				
**Feizi et al [** 4 **]**	11.60*	0.391	0.955	0.669
**Shetty et al [** 3 **]**	11.60	0.297	0.860	0.626
OSI				
**Feizi et al [** 4 **]**	1.850	0.304	0.985	0.637
**Shetty et al [** 3 **]**	1.850	0.216	0.953	0.510
**Golan et al [** 7 **]**	0.671*	0.839	0.450	0.658
PPK				
**Shetty et al [** 3 **]**	25.0	0.811	0.093	0.318
SAI				
**Feizi et al [** 4 **]**	0.895	0.435	0.917	0.644
**Shetty et al [** 3 **]**	0.895*	0.432	0.907	0.656
SDP				
**Feizi et al [** 4 **]**	1.065	0.565	0.857	0.692
**Shetty et al [** 3 **]**	1.065*	0.892	0.814	0.916
SRI				
**Feizi et al [** 4 **]**	0.735	0.826	0.511	0.679
**Shetty et al [** 3 **]**	0.735*	0.676	0.860	0.875
TCP-Central				
**Reddy et al [** 32 **]**	44.12	0.170	0.670	0.510
TCP-Flat				
**Reddy et al [** 32 **]**	43.3	0.830	0.400	0.580
TCP-Steep				
**Reddy et al [** 32 **]**	42.8	0.870	0.360	0.630
Total RMS				
**Reddy et al [** 32 **]**	1.15	0.960	0.580	0.820
Trefoil Z (3,-3)				
**Reddy et al [** 32 **]**	-0.08	0.610	0.580	0.540
Vertical Coma				
**Reddy et al [** 32 **]**	-0.54	0.570	0.900	0.730
3rd-order RMS				
**Reddy et al [** 32 **]**	0.78	0.710	0.900	0.830
4th-order RMS				
**Reddy et al [** 32 **]**	0.37	0.710	0.830	0.790
5th-order RMS				
**Reddy et al [** 32 **]**	0.09	0.670	0.820	0.800
6th-order RMS				
**Reddy et al [** 32 **]**	0.05	0.620	0.780	0.740

Abbreviations: AAI: Asphericity Asymmetry Index; AUC: Area under the Curve; CLMI: Cone Location and Magnitude Index; CSI: Center/Surround Index; DSI: Differential Sector Index; I-S: Inferior-Superior Index; IAI: Irregular Astigmatism Index; KPI: Keratoconus Probability Index; Kprob: Keratoconus Probability; OSI: Opposite Sector Index; PPK: Percentage Probability of Keratoconus; RMS: Root Mean Square SAI: Surface Asymmetry Index; TCP: Total Corneal Power; SDP: Standard Deviation of Corneal Power; SRI: Surface Regularity Index. *denotes cut-off point with best area under the curve if more than one study evaluated index accuracy. Highlighted indices represent the parameters that had an AUC >0.800, which is the minimum diagnostic accuracy recommended for screening of pre-keratoconus.

Therefore, higher values of AAI correlate with increased rates of curvature change and correspond well with the amount of corneal coma [8]. Posterior AAI is a relatively new, manually derived parameter that utilizes BFTA map as well; it is calculated as the absolute value of the highest negative and positive elevations within the posterior corneal zone [7, 9]. While no formal threshold has been set, it is generally accepted that normal posterior AAI values are below 20-25 micrometer (µm) [10]. With the growing consensus that posterior corneal changes may occur first in the development of KC, posterior AAI may be a valuable index to detect pre-keratoconus.

In a recent study, posterior AAI was selected as the most discriminant variable in the detection of suspicious corneas [5]. By using a binary automated decision tree, their study showed posterior AAI to have excellent sensitivity (100%) and specificity (99.5%) in the detection of clinical KC [5]. While diagnostic accuracy decreased slightly for the detection of pre-keratoconus, it was still the most discriminant index among fifty-five Galilei parameters. Interestingly, anterior AAI did not share the same significance in discriminating pre-keratoconus and KC, and in a separate study, anterior AAI did not show significant differences between normal and pre-keratoconic eyes [11]. This finding strengthens the growing consensus that posterior corneal changes occur first in the development of KC [12-17]. 

More recently, no individual index had satisfactory discriminatory accuracy in detecting pre-keratoconus [7]. However, posterior AAI was included in a combination index that had an excellent AUC of 0.960 (sensitivity 90.3%, specificity 92.6%) [9]. While Smadja and colleagues [5] found an optimal cut-off value of 21.5 µm for posterior AAI, Golan et al. [7] found an optimized cut-off of 13.5 µm. These disparities may in part be attributable to each study’s selection criteria and definition of pre-keratoconus. Despite promising initial results, further validation studies with large population datasets are required to affirm the utility of AAI as a reliable screening index.

**Table 4:** The Galilei Clinical “Cheat Sheet”: Suggested Cut-off Values for Keratoconus Indices in Screening Clinical Keratoconus and Pre-Keratoconus

Parameter	Clinical Keratoconus	Pre-Keratoconus
	**Cut-Off Value**	**Cut-Off Value**
CSI	0.70	-
DSI	3.26	-
IAI	0.58	0.450*
I-S	2.33	-
KPI	18.55	-
Kprob	25.55	-
OSI	2.04	-
SAI	1.25	-
SDP	1.93	1.065*
SRI	1.52	0.735*
TCP-Central	45.55	-
TCP-Steep	46.29	-

This table is to serve as a quick screening tool. Formal guidelines are not available at this time and further validation studies are required. Nevertheless, if multiple parameters are calculated to be beyond the recommended threshold values, then it should increase the index of suspicion and trigger further testing for the patient.

Abbreviations: CSI: Center/Surround Index; DSI: Differential Sector Index; I-S: Inferior-Superior Index; IAI: Irregular Astigmatism Index; KPI: Keratoconus Probability Index; Kprob: Keratoconus Probability; OSI: Opposite Sector Index; TCP: Total Corneal Power; SAI: Surface Asymmetry Index; SDP: Standard Deviation of Corneal Power; SRI: Surface Regularity Index. *These are the only indices that demonstrated diagnostic accuracy appropriate for pre-keratoconus (area under the curve >0.800); they do not represent averages as further validation studies are required, however, the authors elected to have them displayed in this table for the convenience of the clinician.

***Center/Surround Index***

**Figure 2:**
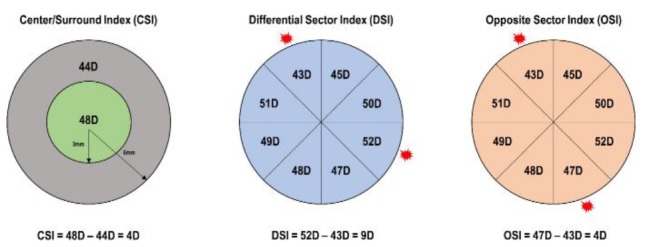
The Center/Surround Index (CSI), Differential Sector Index (DSI), and Opposite Sector Index (OSI) are Calculated based on Eight Arbitrary 45-Degree Sectors of the Corneal Surface. For each Sector Mean Axial Power is Calculated. Sectoral Patterning of the Corneal Surface is Repeated until each Possible Pattern of Sector Distribution is Applied. CSI is the difference of the Area-corrected Corneal Power between a Central 3mm Ring and a 6mm Annulus that Surrounds the Central Ring. DSI is the Greatest difference between any Two Sectors. OSI is the Greatest difference between any Two Opposite Sectors

**Figure 3:**
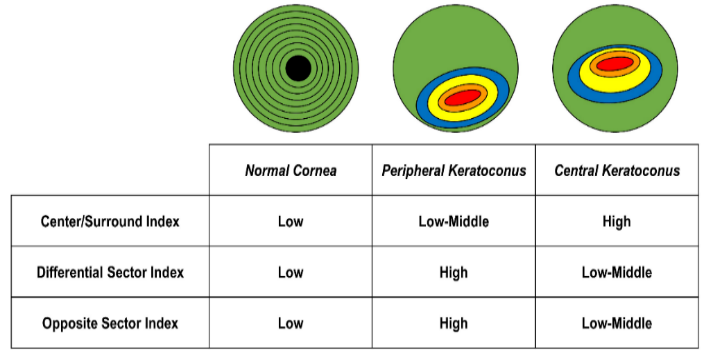
Relationship between Normal Corneal Surface, Astigmatism, and Steepening with Keratoconus Predictive Indices

The Center/Surround Index (CSI) is a quantitative index that characterizes the difference in corneal power between two areas of the cornea. To fully understand CSI, the corneal surface is first divided into eight arbitrary sectors measuring a 45° angle as shown in Fig. 2. For each sector the mean axial keratometric power is then calculated. This process repeats itself until each possible pattern of sector distribution has been applied to the corneal surface [18-20]. Described succinctly by Koch et al., this means that if keratometry is analyzed for 256 semimeridians, then there are 32 possible patterns (256 semimeridians/8 sectors: 32 patterns) [19]. Based on the 45° sectors, CSI represents the difference in the average area-corrected corneal power between a central area (3.0-mm diameter) and an annulus surrounding the central area (3.0-6.0mm diameter) [6]. In normal eyes or with regular astigmatism CSI values are low while in keratoconus CSI is high, thus making CSI a sensitive index for identification of centrally located steepening (Fig. 3) [21, 40]. 

Maeda and colleagues were one of the first to report CSI as a valuable index in differentiating among normal corneas and KC [40]. Moreover, CSI has a significant correlation with visual function, making it a valuable parameter in assessing overall acuity [10]. CSI obtained by Galilei had an excellent diagnostic accuracy in distinguishing keratoconic eyes [6]. Feizi and colleagues reported an AUC of 0.951 for CSI [4]. However, CSI failed as an individual parameter in discriminating pre-keratoconus with a best AUC of 0.534 [4]. These findings were similar to the study by Shetty et al., which reported CSI as a reliable index for identifying KC, but an insensitive diagnostic test for the detection of pre-keratoconus cases [3]. Therefore, based on the available literature we conclude that CSI is a reliable parameter for the diagnosis of KC but should not be used as a standalone index when diagnosing pre-keratoconus. 

***Differential Sector Index***

The differential sector index (DSI) is an index related to the degree of asymmetry present on the corneal surface [19]. As with CSI, DSI is calculated based on the 8-sector pattern of the corneal surface (Fig. 2). With this in mind, DSI characterizes the maximum difference between any two sectors. Thus, with any increase in surface irregularity, the DSI expectedly increases. DSI is a sensitive index for the identification of peripherally located steepening and in corneas with regular astigmatism as shown in Fig. 3 [40].

The studies that evaluate DSI are limited. Similar to other Galilei parameters, DSI is a highly accurate index in the identification of clinical KC [3, 4, 6]. However, in the current literature, DSI is not reported to be a reliable parameter for the identification of pre-keratoconus. Studies have reported AUC <0.650, which is not suitable for clinical standards [3, 4]. Given the available literature, we make the same recommendation for DSI as we did for CSI: an excellent parameter for use in clinical KC, but the index should not be relied on individually for diagnosis of pre-keratoconus. 

***Higher Order Aberrations and Wavefront Analysis***

While higher order aberrations (HOAs) are available in a multitude of topography systems, the Galilei system has demonstrated excellent and perhaps superior repeatability with these measurements [22, 41-43]. Wavefront data from corneal surface analysis is expressed in the form of Zernike polynomials [23-26], and each corneal surface is expressed as the sum of Zernike polynomials. These are generally referred to as corneal aberrations. The Galilei system automatically computes corneal wavefront data and HOAs based on the corneal elevation profile. When compared to single Scheimpflug systems, the Galilei system has less variability in reported Zernike terms [42], likely due to the added benefit of having a dual-channel camera. Another benefit of using the Zernike expansion is that the coefficient of each mode represents the root mean square (RMS) wavefront error that is attributable to that particular mode [26]. Thus, larger coefficient values are associated with modes that contribute a greater amount to the total RMS of the system. 

**Figure 4:**
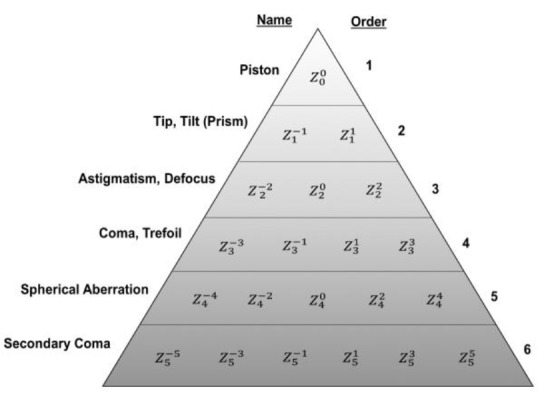
Wavefront Aberrations as Represented by a Zernike Expansion, which is a Weight Sum of Zernike Polynomials where each Polynomial Corresponds to an Aberration Coefficient. Wavefront Aberrations Quantify the Length between a Plane Wavefront Entering the Pupil and the Corresponding Wavefront of Light that Exits a Point on the Retina

The total HOAs are conveniently displayed in the Wavefront Report, which is automatically calculated by the Galilei system. An example of the Zernike polynomial expansion is shown in Fig. 4. The second-order corneal aberrations are the Zernike coefficients for astigmatism and defocus [2, 26]. Third-order corneal aberrations represent the vertical and horizontal values for trefoil and coma [2, 26]. Coma is a valuable indicator for the progression of KC and has been documented as an index that reflects early changes in the corneal surface [27]. Moreover, higher values for vertical coma and large coma RMS values are associated with KC and possible pre-keratoconus [13, 27-31]. Fourth-order HOAs include the quatrefoil and 4th order astigmatism values, which also tend to increase in magnitude if the cornea is deformed [26, 27, 29]. The most clinically relevant fourth-order aberration is the spherical aberration (SA) as it is documented to have a normative range of +0.15 µm to +0.30 µm [2]. Therefore deviations in this range may indicate variations of disease.

Given the variation of HOAs in corneal disease, several studies have explored its value as a screening parameter for KC. Reddy and associates identified several parameters capable of serving as discriminant indices, which included 3rd order, 4th order, 5th order, 6th order, and total higher-order RMS [32]. Furthermore, the RMS for 3rd order, 4th order, 5th order, 6th order, and total higher-order RMS were found to be significantly different between normal eyes and pre-keratoconus [32]. However, only 3rd order and total RMS had acceptable AUC values of 0.830 and 0.820, respectively, in differentiating these two patient groups. Interestingly, in a recent study by Golan and colleagues, none of the HOAs were significantly different between normal eyes and pre-keratoconus [7]. As noted before, it is possible that these disparate conclusions are attributable to the inclusion criteria for pre-keratoconus defined by each study.

Recently, vertical coma was significantly different between normal and pre-keratoconus corneas [11]. However, they did not analyze AUC or discuss the respective sensitivity and specificity for each parameter. Nevertheless, some reports corroborate the importance of vertical coma in its ability to discriminate pre-keratoconus [16, 28]. Vertical coma was significantly greater in progressive versus non-progressive KC [33]. This may indicate that HOAs play a role in analyzing the evolution of KC over time. Moreover, Tellouck and colleagues found that changes in vertical coma occurred before any changes of the posterior corneal surface [38], which may also point towards the diagnostic utility of HOAs in screening of pre-keratoconus. Subsequent validation studies are required to elucidate the clinical value of vertical coma and other HOAs for screening of corneal refractive surgery candidates. 

***Irregular Astigmatism Index***

**Figure 5:**
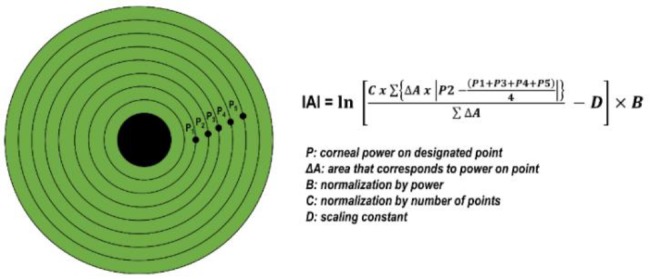
Irregular astigmatism index (IAI) is calculated as the average sum of the inter-ring area-corrected corneal power along every meridian along the corneal surface

The irregular astigmatism index (IAI) describes variation in measured axial power between central rings along any given meridian [19]. IAI is calculated as the average sum of area-corrected keratometric power variations along every meridian for the entire analyzed corneal surface (Fig. 5) [40]. IAI had excellent diagnostic accuracy for KC in multiple studies [3, 4, 6, 9].

For pre-keratoconus, there is no consistent agreement in the literature. Feizi and colleagues reported an AUC of 0.664 [4], which is very similar to the AUC of 0.625 [7]. However, Shetty and associates found a much higher AUC of 0.858 despite using a similar cut-off value as shown in Table 3 [3]. As all three studies were conducted in various regions, it is also possible that inherent population demographics may also play a role in this difference. Thus, our recommendation is to use IAI with caution for pre-keratoconus and to consider a combination of indices when making screening refractive surgery candidates. As with other parameters, IAI as an individual index is capable of distinguishing KC according to the current literature.

***Inferior-Superior Index***

First described by Rabinowitz, the inferior-superior (I-S) index characterizes the dioptric asymmetry between the inferior and superior hemispheres [44]. Rabinowitz and McDonnell modified the threshold values and established criteria that stated an I-S index ≥ 1.4 diopter (D) is susceptible to KC [34]. The I-S index obtained by Galilei is a valuable diagnostic parameter for KC [3, 4, 6]. For pre-keratoconus, however, the diagnostic accuracy is weak which points towards the limitation of the index in diagnosing these cases. Interestingly, however, for pre-keratoconus Shetty et al. reported a mere 10.8% sensitivity with a 90.7% specificity [3]. Thus, while an ideal screening parameter has a higher sensitivity to rule-out disease, the I-S index may be better equipped to rule-in ectatic disease based on its relatively greater specificity. 

***Opposite Sector Index***

As previously described, the opposite sector index (OSI) is calculated based on the 45° sector patterns applied to the corneal surface. OSI is equal to the greatest difference between any two opposite sectors (Fig. 2) [6, 36]. Maeda and Klyce were the first to demonstrate the value of OSI and described its sensitivity in identifying peripherally located corneal steepening (Fig. 3) [36].

Demir et al. were the first to describe the diagnostic accuracy of OSI for KC [6]. Feizi et al. corroborated these findings with a similar AUC, as noted in Table 2 [4]. More recently, Shetty and associates further validated OSI as an index for frank KC [3]. However, in all three studies, OSI was not a reliable parameter for distinguishing eyes with pre-keratoconus. Therefore OSI, while a valuable screening index for KC, should not be used alone when considering pre-keratoconus. 

***Surface Asymmetry Index***

The surface asymmetry index (SAI) is determined by the differences in keratometric power between opposite points distributed on 128 meridians [19]. In simpler terms, SAI characterizes the average of differences in corneal power. Multiple studies have validated the diagnostic utility of SAI in distinguishing clinical KC, which is shown in Table 2 [3, 6]. In the prospective cohort study by Feizi et al., SAI was identified as the best parameter to discriminate clinical KC among all Galilei indices [4]. While as a stand-alone index, SAI was not able to discriminate pre-keratoconus, it was able to do so alongside posterior best-fit sphere in a two-step tree analysis (100% sensitivity, 91.3% specificity) [4]. Given these findings, SAI may play a future role in the diagnosis of pre-keratoconus through a multivariate index. 

***Surface Regularity Index***

The surface regularity index (SRI) characterizes the local irregularities of the corneal surface. SRI is calculated as the sum of power variation along 256 semimeridians on ten central rings over the corneal surface [19]. Thus, similar to DSI, any increase in corneal surface irregularity will manifest as an increase in SRI. Conversely, if the SRI value is equal to zero, the corneal surface is perfectly smooth [6]. SRI was first described by Wilson and Klyce and represents the localized fluctuations of the corneal surface [35]. While there are no formally described ranges, any value of SRI below 1.55 is generally accepted as normal [13, 45].

Galilei computed SRI has excellent diagnostic sensitivity and accuracy for identification of clinical KC (Table 2) [4, 6]. Shetty and colleagues confirmed the diagnostic utility of SRI in their recent study; interestingly, they reported an AUC of 0.875 for detecting pre-keratoconus, which is the highest reported for SRI in the available literature [3]. This is comparable to the Belin-Ambrósio Enhanced Ectasia Display Total Deviation Value (BAD_D) available on the Pentacam system. While a large amount of published data exists for BAD_D, there is inadequate validation for SRI. Studies are required to extrapolate the use of SRI for pre-keratoconus similar to BAD_D.

***Total Corneal Power and Standard Deviation of Corneal Power***

The Total Corneal Power (TCP) represents the average of corneal power for every detected point in a selected region of interest. In order to calculate TCP, the Galilei system utilizes ray tracing of the anterior surface, posterior surface, and pachymetry data [2]. Moreover, TCP can be calculated as the average TCP over steep (TCP-steep) and flat (TCP-flat) meridians, or the average over a central 4.0-mm zone (TCP-central). SDP is simply the value of the standard deviation of TCP. TCP has excellent reproducibility and is a reliable parameter when assessing patients for refractive surgery [42]. Even after surgical procedures, it has been documented that Galilei maintains high intra-observer repeatability for corneal power [46].

TCP-steep was also shown to have an excellent AUC of 0.990 in differentiating KC [32]. This was closely followed by TCP-central with an AUC of 0.94 [32]. Interestingly, however, TCP-flat had only a fair AUC of 0.790. For pre-keratoconus, the AUCs decreased markedly and were weak discriminant parameters [32]. These findings are similar to those of Demir et al., who also concluded that TCP has excellent diagnostic accuracy in distinguishing frank KC as demonstrated in Table 2 [6]. Feizi et al. also concluded that all TCP parameters have excellent diagnostic ability, but that TCP-steep had the highest AUC of 0.994 (94.4% sensitivity, 99.0% specificity) [38]. SDP has also been shown to have high sensitivity for identifying KC [3, 4]. In the recent study, SDP had excellent diagnostic accuracy as an individual parameter in identifying pre-keratoconus (Table 3) [3]. In fact, it was superior to the BAD_D of Pentacam, which had an AUC of 0.887. These studies underline the importance of further exploration of Galilei computed TCP and SDP for screening of refractive surgery candidates and detection of KC.

**Keratoconus Probability Indices**

***Cone Location and Magnitude Index***

**Figure 6:**
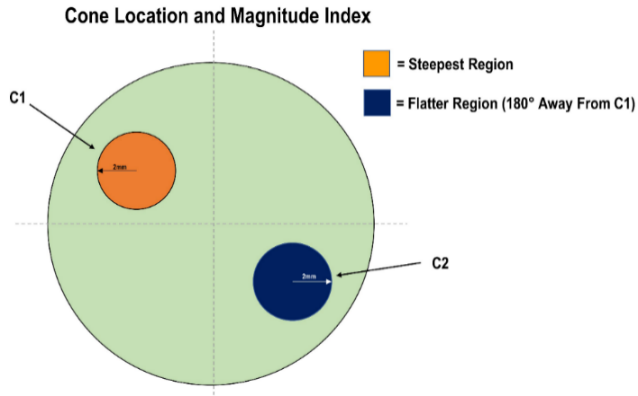
Schematic Representation of the Cone Location and Magnitude Index (CLMI) [46,48] . A first Circle (C1) is 2 mm in Diameter over the Steepest Region of the Corneal Surface. From this a Curvature difference (M1) is Calculated as the Difference between the Points outside and inside C1. A second circle (C2) is Centered 180-Degrees Away from C1. In the same Fashion as M1, A Second Curvature difference, M2, is Calculated. CLMI is then Calculated based on M1 and M2, which Aims to Characterize the Steepest Area of Curvature Relative to the Rest of the Corneal Surface Map

The Cone Location and Magnitude Index (CLMI) was first described by Mahmoud and Roberts [37]. The CLMI relies on an area-corrected average steepest 2 mm-diameter circle within the central 8 mm-diameter anterior curvature map. From this, a curvature difference, M1, is calculated as the difference between all points outside the circle and all points inside the circle. A second circle that is centered 180 degrees away in angular position is analyzed in the same manner, resulting in curvature difference M2. CLMI is then calculated based on M1 and M2. In much simpler terms, the CLMI characterizes the steepest area of curvature, and the magnitude of the index identifies the difference between the steepest area and the rest of the curvature map [37]. This is schematically represented in Fig. 6. CLMI was developed as an index that could be calculated on different topographic systems. The index has shown good repeatability among various devices [41, 47]. Mauger et al. also calls attention to the value of posterior CLMI, which may serve as a valuable measure of asymmetric corneal steepening [47].

Recently, Mahmoud and colleagues modified the CLMI to include posterior surface and corneal thickness data. This addition improved the sensitivity and specificity of the CLMI index from 89.2% sensitivity and 98.8% specificity to 99.4% and 99.6%, respectively [48]. This improvement emphasizes the underlying theme that a robust, multivariate index is superior to an individual parameter in detecting KC. The validation studies for CLMI on the Galilei system are limited. Based on the available literature CLMI it is an excellent screening index for KC, but inadequate for detection of pre-keratoconus as shown in Tables 2 and 3 [3].

***Keratoconus Prediction Index***

First described by Maeda et al., the Keratoconus Prediction Index (KPI) is a compilation index that describes the percent probability of KC based on analysis of the anterior corneal surface [40, 49]. KPI utilizes tomographic data to characterize the probability of having KC. Parameters included in this multivariate index are simulated keratometry, DSI, OSI, CSI, SAI, IAI, and percent area-analyzed. The formula for KPI is found in Formula 1. Based on the Galilei system, KPI from 0 to 10% corresponds to normal or suspicious corneas; KPI from 20 to 30% corresponds to keratoconic or suspicious corneas, and KPI >30% is indicative of pellucid marginal degeneration (PMD) [2, 3].

KPI was capable of differentiating KC from warpage due to keratoplasty, photorefractive keratectomy, radial keratotomy, and contact lens wear [40]. The discriminant functions of KPI are in large part because it is a multivariate parameter that includes a variety of tomographic data. For KC, Maeda and colleagues were able to differentiate KC with a perfect sensitivity (100%) and excellent specificity (96.0%) [40]. This finding is further supported by recent studies that also report excellent diagnostic accuracy for KC [3, 4].

However, for pre-keratoconus, KPI is largely reported to have a moderate to fair diagnostic accuracy as seen in Table 3 [3, 4]. In contrast, none of the KC probability indices, which includes KPI, were significantly different between normal and pre-keratoconus eyes [7]. Again, it is apparent that further validation studies are required to determine the appropriate role of KPI in screening refractive surgery candidates. As a multivariate index, we are more inclined to recommend KPI for screening purposes; however, given the limited studies available, it should not be used alone in screening patients.

KPI=0.30+0.01(-41.23-0.15*DSI+1.18*OSI+1.49*CSI+4.13*SAI-0.60*SimK1+1.08*SimK2-3.74*IAI+0.10*AA)

Formula 1: Keratoconus Prediction Index (KPI) Formula as Described by Maeda et al [40]. Abbreviations: DSI: Differential Sector Index; OSI: Opposite Sector Index; CSI: Center/Surround Index; SAI: Surface Asymmetry Index; SimK1: Simulated Keratometry 1; SimK2: Simulated Keratometry 1; IAI: Irregular Astigmatism Index.

***Keratoconus Probability***

The Keratoconus Probability (Kprob) is an index that characterizes sensitivity and specificity of the reported KPI based on a normative and keratoconic database [37]. Expectedly, Kprob has a inverse relationship with visual function [10]. Feizi et al. were the first to report the utility of Kprob in their prospective study evaluating clinical KC and pre-keratoconus. They found an excellent AUC of 0.998 for KC, but an inferior AUC of 0.669 when distinguishing eyes with pre-keratoconus [4]. This result was similarly obtained by Shetty and colleagues, who reported an AUC of 0.993 for KC and a similar AUC of 0.626 for pre-keratoconus [3]. For this reason, we recommend the use of Kprob only for discriminating clinical KC.

***Percentage Probability of Keratoconus***

Mahmoud and colleagues were also the first to describe Percent Probability of Keratoconus (PPK), which is defined as the optimal threshold for detecting KC [37]. The PPK is calculated from a validated equation that incorporates CLMI using axial data. The recommended cutoff value for clinical KC is 45.0%, while the cutoff value for pre-keratoconus is 20.0% [37]. Like the other probability indices, PPK can discriminate KC with excellent diagnostic accuracy but fails in distinguishing pre-keratoconus KC from normal eyes [2, 3].

## DISCUSSION

**Application and Interpretation of Galilei Indices**

As with the Pentacam camera, the majority of screening indices on the Galilei system play a valuable role in discerning eyes with KC. When assessing pre-operative risk, it is often the pre-keratoconus cases that are of particular interest. Based on this comprehensive review, physicians should not rely solely on a single parameter to guide the course of treatment or surgical eligibility. Instead, we recommend a step-wise approach that begins with the patient history and incorporates tomographic data. By constructing the bigger clinical picture, clinicians are best able to stratify patients and turn them away from surgery if there is sufficient clinical concern. 

Based on this review, nearly all Galilei calculated KC indices are capable of distinguishing clinical KC with an AUC >0.900. For pre-keratoconus, the only standalone parameter that meets an AUC of 0.900 is SDP. However, as this was only validated in one study, close attention to AAI and SRI is recommended as they have fair to good diagnostic accuracy; however, in the studies thus far these indices have not demonstrated strong enough diagnostic accuracy to be accepted as individual screening parameters. Moreover, given the limited amount of studies, further investigations are required to appreciate the diagnostic utility of these indices in full. Our recommended optimized cut-off values for each index is highlighted in Table 4. Unfortunately, the lack of studies interferes with our ability to recommend pre-keratoconus thresholds which is dissimilar from the other articles in this three-part series. Nevertheless, we have identified the parameters that have demonstrated an AUC >0.800 in Table 3 to draw special attention to the indices that are likely to have the biggest role in future refractive screening.

These recommendations can be used as a quick reference tool for daily clinical practice. For pre-keratoconus, our recommendation is to evaluate the parameters in total. For example, if a patient meets criteria for pre-keratoconus across multiple indices, then the index of suspicion that disease is present should be higher. Studies have shown that the best indices for screening are multivariate parameters that combine pachymetric and tomographic data. We anticipate that future studies will ultimately confirm this and that combination indices will prove to have the highest diagnostic accuracy. 

Another application of the dual Scheimpflug system is its superior accuracy in predicting IOL power prediction [50]. Beyond its role in planning and screening for refractive surgery, the Galilei system has a clinical role in tracking outcomes and refractive error. In a recent study, it was identified as a superior system in evaluating corneal stabilization following corneal crosslinking [51]. The camera has also demonstrated excellent repeatability even after laser ablation [46], and thus its application includes monitoring post-operative changes that may be indicative of pre-keratoconus.

**Limitations**

At present, studies assessing the diagnostic accuracy of Galilei indices are limited and there are inconsistencies in the literature. Validation studies will further elucidate the Galilei indices and their role in screening of the refractive surgery candidates. Most importantly, the ability to accurately diagnose pre-keratoconus, during the screening process will lead to safer and better outcomes. As mentioned in the Pentacam review, creating a universal definition for these particular cases will also facilitate better evaluation of diagnostic indices. 

The majority of published research compares Galilei to other Scheimpflug devices. However, there are varying levels of agreement amongst different devices. While many studies have found good concordance [52-59], others have recommended not to interchangeably use measurements calculated by various devices [60-63]. Further, some studies describe agreement for specific parameters, while finding significant differences for others [54, 64]. Given the disparate findings in the literature, there is insufficient data to conclude the devices’ measurements are interchangeable at this time. Even though systems like Pentacam and TMS-5 operate on the same Scheimpflug principles, the devices cannot reliably be used interchangeably. Development of indices that can be used on any device such as CLMI will help reduce the impact of this limitation on future studies. 

While the current review focuses on the KC predictive indices calculated by the Galilei system, it is important to mention the value of elevation data in screening refractive surgery candidates. Galilei computed elevation data still plays an important role when evaluating for KC. As described by Jafarinasab and colleagues, both anterior and posterior elevation can discern clinical KC and pre-keratoconus [65]. Interestingly, their study found that a 3-mm zone discriminates KC, while the 7-mm zone optimally distinguishes pre-keratoconus. This finding may indicate that the early ectatic changes occur in the periphery, yet as the disease progresses, the central involvement becomes more prominent.

**Looking Ahead**

Recent studies indicate that combination indices have the highest diagnostic accuracy [9, 66]. It is likely that the future will include these metrics for screening the refractive surgery patient. As with Pentacam, we do not recommend the use of an individual index for discerning KC. Instead, the best approach in the clinical setting is to evaluate multiple indices. While individual indices, such as AAI, have diagnostic utility, the lack of validation studies hinders our ability to recommend a single index for refractive screening. 

The additive value of optical coherence tomography and ultrasound assessments can lead to improved diagnostic accuracy of combination parameters [66]. Biomechanical evaluation when evaluating ectasia risk can be incorporated into screening. With the advent of technologies that assess biomechanical properties, the armamentarium of screening methods continues to grow [67]. While we address biomechanical properties independently in the third article of this series, the highest yield screening method is one that combines all available corneal surface data. 

There are already many studies that have demonstrated the value of applying neural networks, and this is likely where the future of refractive screening is headed [4, 5, 9, 49]. While it will be decades until both patient and physician are comfortable with automated analysis, the application of artificial intelligence, specifically deep learning algorithms, may prove to be the best approach for diagnosing pre-keratoconus. Nevertheless, the value of clinical picture cannot be overstated. The refractive indices provided by Galilei and other Scheimpflug analyzers are best interpreted in the clinical context.

## CONCLUSION

Corneal surface indices provided by the Galilei system are reliable parameters in the identification of keratoconus. However, as evidenced by our review, there is insufficient data to conclude the reliability of these parameters in screening of pre-keratoconus. In the first article of this series we arrived at a similar conclusion for the Pentacam system. When comparing these two camera systems, the literature is far more robust for the Pentacam camera. Thus, future investigations are required to determine the true diagnostic accuracy of Galilei indices in the diagnosis of pre-keratoconus. Based on the current literature, the best approach for screening of the refractive surgery candidate is a combination of refractive indices aimed towards prevention of pre-keratoconus and postoperative iatrogenic ectasia. In the third article of this series, the authors introduce biomechanical parameters that can also be used in supplementing corneal surface data. Ultimately, the greatest diagnostic accuracy is expected from a parameter that combines pachymetric, keratometric, elevation, and biomechanical data from various modalities of corneal imaging. 

## DISCLOSURE

Ethical issues have been completely observed by the authors. All named authors meet the International Committee of Medical Journal Editors (ICMJE) criteria for authorship of this manuscript, take responsibility for the integrity of the work as a whole, and have given final approval for the version to be published. No conflict of interest has been presented. Funding/Support: None
